# Expression Evolution of Ancestral XY Gametologs across All Major Groups of Placental Mammals

**DOI:** 10.1093/gbe/evaa173

**Published:** 2020-08-13

**Authors:** Mónica Martínez-Pacheco, Mariela Tenorio, Laura Almonte, Vicente Fajardo, Alan Godínez, Diego Fernández, Paola Cornejo-Páramo, Karina Díaz-Barba, Jean Halbert, Angelica Liechti, Tamas Székely, Araxi O Urrutia, Diego Cortez

**Affiliations:** e1 Center for Genome Sciences, UNAM, Cuernavaca, Mexico; e2 Center for Integrative Genomics, University of Lausanne, Switzerland; e3 Milner Centre for Evolution, Department of Biology and Biochemistry, University of Bath, Claverton Down, Bath, United Kingdom; e4 Ecology Institute, UNAM, Mexico

**Keywords:** placental mammals, sex chromosomes, Y chromosome, gene expression levels, dosage compensation mechanisms

## Abstract

Placental mammals present 180 million-year-old Y chromosomes that have retained a handful of dosage-sensitive genes. However, the expression evolution of Y-linked genes across placental groups has remained largely unexplored. Here, we expanded the number of Y gametolog sequences by analyzing ten additional species from previously unexplored groups. We detected seven remarkably conserved genes across 25 placental species with known Y repertoires. We then used RNA-seq data from 17 placental mammals to unveil the expression evolution of XY gametologs. We found that Y gametologs followed, on average, a 3-fold expression loss and that X gametologs also experienced some expression reduction, particularly in primates. Y gametologs gained testis specificity through an accelerated expression decay in somatic tissues. Moreover, despite the substantial expression decay of Y genes, the combined expression of XY gametologs in males is higher than that of both X gametologs in females. Finally, our work describes several features of the Y chromosome in the last common mammalian ancestor.

**Significance**

Previous studies unveiled many features of the evolution of X and Y chromosomes in placental mammals. Nevertheless, the general patterns of gene expression evolution of XY gametologs across placental species remain largely unexplored outside humans and rodents. To close this knowledge gap, in this study we explored the conservation, expression evolution, and dosage compensation mechanisms of ancestral XY gametologs across all major groups of placental mammals. We uncovered important evolutionary processes that have shaped the expression patterns of sex chromosomes in the last mammalian ancestor.

## Introduction

Sex chromosomes in marsupial and placental mammals originated from a pair of autosomes (Bachtrog 2013) following the emergence of the sex-determining gene *SRY* (Sinclair et al. 1990) on the proto-Y chromosome. The protein coded by the *SRY* gene was capable to directly regulate the expression of *SOX9* (Sekido and Lovell-Badge 2008; Li et al. 2014) and, therefore, initiate the signaling cascade to develop testis. The origin of the sex chromosomes in therians occurred ∼180 Ma (Cortez et al. 2014) and, since then, recombination has been suppressed along the majority of the X and Y chromosomes (Lahn and Page 1999), giving rise to the male-specific region of the Y chromosome (MSY) (Skaletsky et al. 2003). Consequently, lack of homologous recombination prompted the rapid loss of genetic content of the Y chromosome (Charlesworth B and Charlesworth D 2000; Hughes et al. 2012) and the evolution of mechanisms that could restore expression balance between males (one active X chromosome) and females (two active X chromosomes). Dosage balance on the X chromosome was later achieved by the recruitment of a long noncoding RNA (*Xist*) capable of mediating the inactivation of one X chromosome in females (Plath et al. 2002).

Lack of homologous recombination between Y and X chromosomes also allowed the proliferation of repeated sequences on the Y chromosome (Skaletsky et al. 2003). Accumulation of a large number of duplicated elements (Bachtrog 2013) resulted in complex genomic sequences, which are difficult to analyze and assemble. Several Y chromosomes have been sequenced though: human (Skaletsky et al. 2003), chimpanzee (Hughes et al. 2005, 2010), gorilla (Tomaszkiewicz et al. 2016), macaque (Hughes et al. 2012), mouse (Soh et al. 2014), pig (Skinner et al. 2016), and horse (Janecka et al. 2018). Specific efforts also recovered Y genes from cat and dog (Pearks Wilkerson et al. 2008; Li et al. 2013), bull and marmoset (Bellott et al. 2014), and some Y scaffolds in the polar bear (Bidon et al. 2015), the gray wolf (Smeds et al. 2019) and the red fox (Rando et al. 2019). Also, a subtraction approach developed to work with male and female transcriptome data allowed the reconstruction of Y-linked gene repertoires in ten species of placental, marsupial, and monotreme mammals (Cortez et al. 2014).

Thus far, analyses of the MSY in placental mammals have revealed the presence of large palindromic structures (Skaletsky et al. 2003), Y-linked genes conserved across species (Hughes et al. 2012; Cortez et al. 2014; Janecka et al. 2018), presence of ampliconic gene families with large number of copies (Goto et al. 2009; Soh et al. 2014; Ghenu et al. 2016; Brashear et al. 2018; Vegesna et al. 2019), gene functions related to spermatogenesis (Colaco and Modi 2018; Liu 2019), and retention of ancestral X–Y gene pairs (i.e., XY gametologs) that are under purifying selection (Wilson and Makova 2009), code for proteins with regulatory functions and are haploinsufficient (Bellott et al. 2014; Cortez et al. 2014).

Haploinsufficiency means that the expression level of one copy does not produce sufficient protein to realize a biological function. We expect, therefore, some functional redundancy between X and Y gametologs in males to overcome haploinsufficiency, whereas the expression of an active X gametolog is complemented (at least in humans and mouse) by the expression of an X gametolog that escapes X chromosome inactivation in females (Bellott et al. 2014; Tukiainen et al. 2017). Notably, expression levels of X-linked genes in humans, including X gametologs, are affected by the presence of multiple copies of X and Y chromosomes (Raznahan et al. 2018), indicating dynamic coexpression networks between sex chromosomes that regulate gene expression.

Several studies have examined the evolutionary constraints and expression of X- and Y-linked genes in primates and mouse (Lingenfelter et al. 2001; Xu and Disteche 2006; Isensee et al. 2008; Xu et al. 2008; Wilson Sayres and Makova 2013; Slavney et al. 2016; Godfrey et al. 2020). Nevertheless, the general patterns of gene expression evolution of XY gametologs across major placental groups have remained largely unexplored. In this study, we expanded the number of species with Y-linked genes by recovering Y gametologs from ten additional species of placental mammals. We used extensive RNA-seq data from 17 species from all major groups of placental mammals to unveil the expression evolution of ancestral XY gametologs, evaluated potential dosage compensation mechanisms, and defined attributes already present on the Y chromosome of the mammalian ancestor.

## Materials and Methods

### Data Collection

We collected published RNA-seq data for nine placental mammals: human, macaque, rabbit, mouse lemur, sheep, panda, rat, mouse, and marmoset (supplementary table 1, Supplementary Material online). Also, we generated strand-specific RNA-seq libraries (using the Illumina TruSeq Stranded mRNA Library protocol) for eight placental mammals: tree shrew, hamster, guinea pig, cow, pig, hedgehog, armadillo, and tenrec; at least one female and one male brain/cerebellum, heart, liver, kidney, and gonads. Each library was sequenced on Illumina HiSeq 2500 platforms at the Lausanne Genomic Technologies Facility. Libraries were sequenced as 100-nt single end with 34 million reads on average per sample and >93% of reads with *Q* > 35 of mean quality (supplementary table 1, Supplementary Material online). We also generated extra RNA-seq data for sheep. Besides, we generated male DNA-seq libraries for seven placental species: hamster, guinea pig, rabbit, sheep, hedgehog, armadillo, and tenrec using the Illumina TruSeq DNA protocol for short insert size (target size 400–450 nt). We sequenced 100-nt paired-end DNA-seq libraries on an Illumina HiSeq 2500 sequencers (112 million reads on average). For mouse lemur, panda, and tree shrew, we worked with the available genomic published data (supplementary table 1, Supplementary Material online). We also collected published RNA-seq data for three outgroup species: chicken, platypus, and *Anolis carolinensis* (supplementary table 1, Supplementary Material online).

### Reported Y-Linked Genes

We collected the nucleotide sequences of ancestral Y-linked genes (or transcripts) from human, macaque, marmoset, mouse, rat, guinea pig, rabbit, cow, sheep, panda, pig, and hedgehog directly from scientific publications, the Ensembl database (release 96; www.ensembl.org; last accessed August 21, 2020) or the NCBI-GenBank (www.ncbi.nlm.nih.gov/genbank; last accessed August 21, 2020) database (supplementary table 2, Supplementary Material online).

### Assembly of Y-Linked Transcripts

To assemble Y-linked transcripts in placental mammals, we used a subtraction approach based on male/female RNA-seq data. Subtraction approaches have been used to find sex-linked sequences in mammals/birds (Cortez et al. 2014), the green anole *A. carolinesis* (Marin et al. 2017), casque-headed lizards (Acosta et al. 2019), water skinks (Cornejo-Paramo et al. 2020), plants (Akagi et al. 2014), and insects (Carvalho and Clark 2013). This method allows the recovery of nucleotide sequences and the identification of sex-linked genes that are necessary for evolutionary and functional analyses. We used the subtraction approach to recover Y-linked transcripts in mouse lemur, tree shrew, hamster, guinea pig, rabbit, sheep, panda, hedgehog, armadillo, and tenrec. Specifically, for each species, we first removed reads with ambiguous nucleotides (N). Next, we concatenated male RNA-seq reads from male tissues into one file and aligned these reads using Hisat2 (default options; v2.0.2) (Kim et al. 2015) against both the female reference genome downloaded from the Ensembl database (release 96; www.ensembl.org; last accessed August 21, 2020) and the de novo reconstructed female transcriptome; reads not mapping were selected; female transcriptomes were obtained using female tissues (brain/cerebellum, kidney, heart, liver, and ovary) with Trinity (v2.0.2, default options). We also used the female RNA-seq data to build an index of 35-bp k-mers for each species, following a previous procedure (Akagi et al. 2014). We calculated the frequency of these 35-bp k-mers and removed those showing frequencies below ten; we did not consider rare k-mers as part of the overall signature of the female transcriptome. We used Bowtie2 (2.1.0) (Langmead and Salzberg 2012) to align the more abundant 35-bp k-mers to the male reads that did not align with the female transcriptomes (with no mismatches and no indels allowed); we selected those male reads with no successful alignments. With the few remaining reads, we assembled a male-biased transcriptome with Trinity (v2.0.2, default options) (Grabherr et al. 2011). We obtained around 20,000 transcripts per species showing biased expression in male tissues. These transcripts with male-biased expression could belong to autosomes or the X chromosome, and may not necessarily represent Y-linked genes. To test for Y-linked sequences, we used BlastN (Altschul et al. 1990) to perform sequence searches in male/female genomic data. Female genomic reads were downloaded from NCBI (www.ncbi.nlm.nih.gov; last accessed August 21, 2020); raw reads from the female reference genome projects. Moreover, we produced male genomic data for specific species. Male transcripts were marked as Y-linked when showing 100% identity over 90% or more of their sequence length aligned against the male genomic data and no significant alignments against the female genomic data. Transcripts present in both the male transcriptome and the male genome were considered as Y-linked. These Y-linked sequences were further examined based on alignments with Muscle (Edgar 2004) of X–Y pairs in each species and Y–Y alignments across placental species. Recovering ancestral Y gametologs using subtraction-based approaches is easier because these genes display a larger number of nucleotide differences with X gametologs. Note that subtraction methods are more accurate when the Y genes show an overall nucleotide sequence similarity below 98% compared with the X gametolog nucleotide sequence (Cortez et al. 2014). Consequently, recent Y-linked genes on the MSY may not be identified. Group- and species-specific Y transcripts were not included in this study. As an additional control, we ran the subtraction-based method with male/female RNA-seq data from human, marmoset, mouse, rat, pig, cow, and opossum, for which Y sequences have been obtained using targeted direct-sequencing methods. The results from the sequence comparison are detailed in supplementary table 3 and the sequence alignments are in Supplementary Material online.

### Defining Y-Linked Gene Absence

In placental species with sequenced Y chromosomes, we defined the absence of an Y gene when it was not annotated on the Y chromosome and BlastN/tBlastN searches using orthologous Y-linked genes from other species did not retrieve significant matches. For species without sequenced Y chromosomes, we searched male genomic reads with BlastN and tBlastN using orthologous Y-linked genes from other species as queries. When sequence searches retrieved matches for the X gametolog instead of the Y gametolog, we considered the Y gene to be absent. We did not assess species for which male genomic data were not available or were insufficient.

### Identification of Y and X Gametolog Identities

To establish Y gene identity, we searched NCBI GenBank (www.ncbi.nlm.nih.gov/genbank/; last accessed August 21, 2020) with BlastN and BlastX for the closest homologs in the Mammalian taxa and selected transcripts that coded for known Y- or X-linked proteins. BlastX searches also allowed the identification of CDS regions. Other Y-linked sequences found, often representing pseudogenes, transposable elements, or potential long noncoding RNAs, were not considered in downstream analyses. Then, the best BlastN match (usually around 92–95% identity over the entire sequence) onto the annotated X chromosome of the reference genomes was considered the X gametologs. Some reference genomes lacked annotations for X gametologs (*RBMX* in macaque; *DDX3X* in tree shrew; *USP9X* in the guinea pig; *TMSB4X* in sheep; *RPS4X*, *RBMX*, *ZFX* in hedgehog; *RPS4X*, *RBMX*, *TSPLY2*, *TMSB4X*, and *ZFY* in tenrec). In these specific cases, we included in the analyses the transcripts for the X gametolog from the complete transcriptome reconstruction using female tissues.

### Identification of Retrogenes

**Fig. 1.**
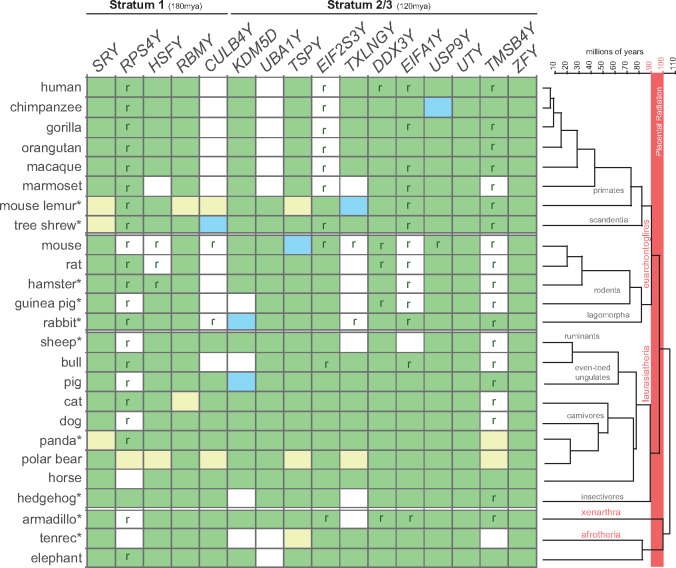
Distribution of ancestral Y genes in 25 placental mammals. Green squares represent the presence of a Y gene, whereas white squares represent the absence of a Y gene. Blue squares denote Y genes annotated as pseudogenes but with detectable expression levels. Yellow squares represent Y genes with unclear status due to lack of expression data for *SRY* (mostly expressed during development), lack of testis data and absence of testis-specific genes (*RBMY*, *CULB4Y*, and *TSPY*) for mouse lemur, missing genes in published references (*RBMY* in cat and several Y genes in polar bear), unclear Y-specific transcripts (*TMSB4Y* in panda, *TSPY* in tenrec). The ten species of placental mammals for which Y-linked genes were obtained in this study have their names marked with an asterisk. The presence of XY-derived retrogenes is highlighted with an *r*. The phylogenetic tree highlights the major groups of placental mammals and their divergence time (in millions of years).

Retrogenes were identified following a previous procedure (Marques et al. 2005). Briefly, we retrieved amino acid sequences for X and Y gametologs from the 25 species listed in figure 1. The protein sequences were then used as queries in searches against the 25 complete genomes (Ensembl database, release 96; www.ensembl.org; last accessed August 21, 2020) using tBLASTn; default options (Altschul et al. 1997). Adjacent homology matches were merged, combining only all nearby matches, and we verified they lacked reported introns. We required that query and target sequences had >50% similarity on the amino acid level and over >80% of their length shared. For each species, we aligned retrogenes to the parental X and Y gametologs using PRANK (Loytynoja and Goldman 2005), codon-based option (- codon). Pairwise *d*_S_ values for each of the two following pairs, retrogene—parental X gametologs and retrogene—Y gametolog, were obtained using codeml, implemented in the PAML package; runmode = -2, seqtype = 1, CodonFreq = 2 (Yang 1997). X or Y gametologs showing the lowest *d*_S_ values were considered to be the parental gene for a given retrogene.

### Expression Analyses

**Fig. 2.**
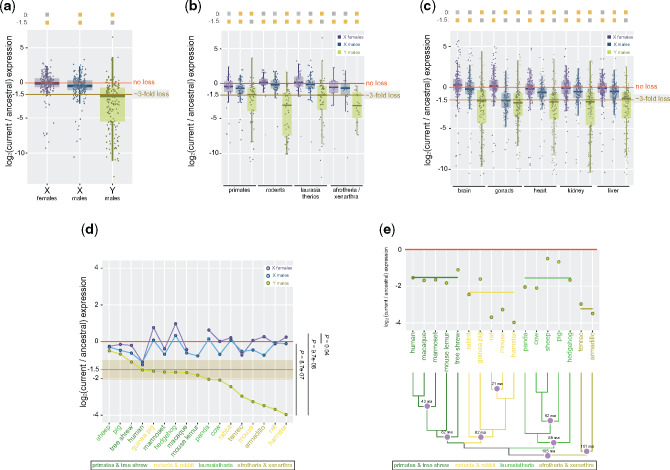
Expression levels of ancestral XY gametologs in 17 placental mammals. (*a*) Boxplots representing the current/ancestral expression ratio of X and Y gametologs in males and females (*n* = 200 genes). (*b*) Boxplots representing the current/ancestral expression ratio of X and Y gametologs in males and females according to the phylogenetic group: *Pr* data from primates including tree shrew (*n* = 60 genes); *Ro* data from rodents including rabbit (*n* = 52 genes); *La* data from laurasiatheria (*n* = 65 genes); *At* data from armadillo and tenrec (*n* = 23 genes). (*c*) Boxplots representing the current/ancestral expression ratio of X and Y gametologs in males and females in brain, gonads, heart, kidney, and liver; data from panda were not included. (*a*–*c*) Significant differences (Mann–Whitney *U* test): Benjamin–Hochberg-corrected *P *<* *0.05 of ratios against a distribution with a fixed median of 0 (i.e., similar expression levels of current and ancestral states) or −1 (i.e., 2-fold loss in expression levels between current and ancestral states). Gray filled squares denote nonsignificant differences, whereas yellow filled squares denote significant differences. Error bars, maximum and minimum values, excluding outliers. (*d*) Dot plot representing median current/ancestral expression ratios of X and Y gametologs in males and females for each species (*n* = 200 genes). Species are ordered on the *X*-axis based on the Y gametologs values (decreasing order). Significant differences (Mann–Whitney *U* test) between groups. (*e*) Dot plot representing the median current/ancestral expression ratio of Y gametologs according to the phylogeny of the species. Colored bars indicate the reconstructed ancestral expression levels for each group. Purple dots on the phylogenetic tree indicate species divergence times. (*a*–*b*) and (*d*–*e*) Median expression levels across tissues (TPM) were used.

We downloaded from the Ensembl (release 96; www.ensembl.org; last accessed August 21, 2020) database the complete transcriptome (cDNA files) for human, macaque, marmoset, tree shrew, mouse lemur, mouse, rat, hamster, guinea pig, rabbit, cow, sheep, pig, panda, hedgehog, armadillo, and tenrec. Ensembl transcriptomes for human, macaque, and pig already included Y-linked transcripts. For all other species, we added to the transcriptome files the Y-linked genes reported in scientific publications, the NCBI-GenBank database or obtained based on the subtraction approach. In specific cases, we added to the files a few X-linked transcripts missing (see above). Then, we aligned RNA-seq reads from brain/cerebellum, heart, kidney, liver, and gonads (testis and ovary) and estimated expression levels as transcripts-per-million using Kallisto (Bray et al. 2016), specifying 100 bootstraps. All single-end libraries were produced at the Lausanne Genomic Technologies Facility (https://wp.unil.ch/gtf/; last accessed August 21, 2020) following the same protocol with an average fragment length of 300 ± 50 bp (information needed to run Kallisto; -l and -s parameters), which were verified using a Bioanalyzer. Given that Y-linked genes from many species were transcripts reconstructed from male RNA-seq data, we reasoned that expression analyses should be carried out at the level of transcripts for all species. XY gametologs show 92–95% identity over the entire sequence, so Kallisto is capable of assigning the correct reads to these genes. We tested for cross-gametolog mapping by in silico generating random RNA-seq reads of 100 nt long based on the nucleotide sequences of X gametologs (100,000 reads) and the nucleotide sequences of Y gametologs (100,000 reads). We mapped the Y- and X-derived reads to the transcriptomes separately. In the hypothetical scenario where cross-gametolog mapping has an important effect, several reads would equally map to both gametologs and, therefore, we would see expression levels of X genes when mapping Y-derived reads and expression of Y genes when mapping X-derived reads. We observed that cross-gametolog mapping is remarkably low in our set of ancestral XY gametologs (supplementary fig. 1, Supplementary Material online). When multiple individuals for males or females were available, we calculated median expression levels per tissue. We also combined median expression levels for the brain and cerebellum. Expression level normalization across samples was performed using a scaling procedure (Brawand et al. 2011) that uses one-to-one orthologous genes expressed in all samples showing the lowest variance across samples (supplementary figs. 2 and 3, Supplementary Material online). RNA-seq data for panda were from particular tissues (tongue, pituitary, colon, etc.) and RNA-seq data for mouse lemur were only available for males; these data were only included in the general Y expression profiles reported in figure 2. Mapping RNA-seq reads to multicopy Y genes is a complex task, particularly, because many multicopy Y genes are 99–100% identical at the nucleotide level. In gene repertoires recovered using transcriptome data and subtraction approaches, we obtained one gene copy representing all copies of a multicopy Y gene. However, for species with fully sequenced Y chromosomes, multicopy Y genes are well annotated and may include between two and ten copies. So, for species with sequenced Y chromosomes, when multiple copies of a Y or X gene were present, we added the individual gene expression levels to obtain one value per gene. We performed the same strategy for retrogenes of a given X or Y parental gametolog when multiple retrogenes for a given parental gene were present in the same species. *SRY* was excluded from the analyses because this gene is primarily expressed during development. We performed a principal component analysis to determine whether samples grouped by tissue (no batch effects present) or experiment (batch effects present). We used expression levels of one-to-one orthologs across species. We found that samples clustered by tissue, which is expected when batch effects are not present (supplementary fig. 4, Supplementary Material online). Comparisons of expression levels between current and ancestral states were carried out as previously described (Julien et al. 2012; Cortez et al. 2014). Specifically, to infer ancestral expression levels we exploited the fact that current sex chromosomes are derived from ancestral autosomes and, therefore, have autosomal counterparts in species with nonhomologous sex chromosomes, which are informative concerning protosex chromosome expression patterns. We calculated ancestral sex chromosome expression levels as median expression levels of autosomal one-to-one orthologs of X genes in outgroup species with different sex chromosomes systems: *A. carolinensis*, platypus, and chicken. Ancestral inferred expression output values were calculated per one gene copy/allele, that is, the obtained values were divided by 2. We obtained 16 ancestral values, one for each XY gametolog pair. These ancestral values were then used against the expression levels of all current XY gametologs from the different placental species. Furthermore, we only analyzed the expression level of X gametologs in a given species when Y gametologs were present. The tissue specificity index (TSI) for a given gene was calculated as the expression level (Transcripts Per Million [TPM]) in the tissue with the highest expression level divided by the sum of expression values in all tissues (Julien et al. 2012). Expression values are listed in supplementary table 4*a*–*d*, Supplementary Material online. Genes used in the different analyses are listed in supplementary table 5*a*–*e*, Supplementary Material online. Furthermore, we verified the general trends of Y expression loss using two alternative metrics: Fragments Per Kilobase of exon model per Million reads mapped values obtained from Hisat2 (default options; v2.0.2)–Cufflinks (default options; v2.2.1) (Trapnell et al. 2013; Kim et al. 2015) and normalized using one-to-one orthologs and a scaling procedure (Brawand et al. 2011), and raw count obtained from Kallisto (Bray et al. 2016) and normalized using one-to-one orthologs and the Trimmed Mean of M-Values normalization in the EdgeR package (Robinson et al. 2010) (supplementary figs. 5 and 6, Supplementary Material online).

### Statistical Analyses

All statistical analyses were performed using the R package, standard libraries. Data were plotted using the R package, “ggplot2” library (https://ggplot2.tidyverse.org/; last accessed August 21, 2020). We calculated significant differences using the nonparametric Mann–Whitney *U* test, which was corrected for false discovery rate using the Benjamin–Hochberg correction at 0.05. We tested current/ancestral ratios and female/male ratios against theoretical distributions with either a fixed median of 0 (i.e., similar expression levels in current and ancestral states or between males and females) or −1 (i.e., lower expression levels in current vs. ancestral states or males vs. females). Ancestral state reconstruction was calculated in the R package using the libraries: *phytools*, *ape*, and *maps*. Life-history traits for placental mammals are reported in the supplementary table 6, Supplementary Material online.

## Results

### Conservation of Ancestral Y Gametologs across 25 Species of Placental Mammals

Previous studies reported Y-linked genes in 15 species of placental mammals (fig. 1) (Skaletsky et al. 2003; Hughes et al. 2005, 2012; Li et al. 2013; Bellott et al. 2014; Cortez et al. 2014; Soh et al. 2014; Bidon et al. 2015; Skinner et al. 2016; Tomaszkiewicz et al. 2016; Janecka et al. 2018). We recovered Y-linked genes from ten additional species of placental mammals (fig. 1, names marked with an asterisk; supplementary tables 1–3, Supplementary Material online) based on a subtraction approach that uses male and female RNA-seq data (see Materials and Methods). We worked with species from lineages that were not previously explored, such as lemurs (primates), tree shrew (scandentia), rabbit (lagomorpha), hedgehog (insectivores), armadillo (xenarthra), and tenrec (afrotheria). Ultimately, we compiled a catalog of Y gametologs across 25 species covering the four major groups of placental mammals: euarchontoglires, laurasiatherian, xenarthra, and afrotheria (fig. 1).

We focused the analyses on the ancestral Y gametologs that originated ∼180 (stratum 1) and ∼116 (stratum 2) Ma (Cortez et al. 2014) before the radiation of the four major groups of placental mammals, which took place 90–100 Ma (estimates retrieved from the TimeTree database; www.timetree.org; last accessed August 21, 2020).

Earlier work (Lahn and Page 1999; Hughes et al. 2012; Li et al. 2013; Cortez et al. 2014; Janecka et al. 2018) identified 16 ancestral Y gametologs from strata 1 to 2 on the Y chromosomes of placental mammals (fig. 1). Our analyses in ten additional species, from five new orders, identified the same 16 ancestral gametologs, although we did not limit the analyses to these specific genes (see Materials and Methods). This result indicated that the ancestor of placental mammals already presented a reduced and very distinctive set of genes on its MSY.

We observed that Y gametologs have followed different evolutionary trajectories across the 25 species of placental mammals (fig. 1). Seven of them are present in all the analyzed species (*SRY*, *RBMY*, *TSPY*, *DDX3Y*, *UTY*, *USP9Y*, and *ZFY*), whereas others seem to have been lost one (*EIF2S3Y*), two (*HSFY*, *UBA1Y*, *EIFA1Y, CULB4Y*), three (*TXLNGY*), four (*KDM5D*), or five times (*RPS4Y* and *TMSB4Y*). From the distribution of Y gametologs across the species’ phylogeny, we could not distinguish a clear pattern of why some Y genes are more frequently lost than others. We, therefore, explored the possibility that expression level changes could play an important role in the loss of Y-linked genes.

### Expression Evolution of XY Gametologs in 17 Species of Placental Mammals

We collected or generated RNA-seq data for 17 species of placental mammals and estimated the expression levels for X and Y gametologs in males and females (supplementary table 1, Supplementary Material online). We first analyzed whether XY gametologs, overall, experienced changes in expression levels following the recombination arrest between X and Y chromosomes. To do so, we compared for each pair of XY gameologs the current expression levels (median value across tissues) against ancestral expression levels (the expression on the protosex chromosomes). To obtain ancestral expression levels, we exploited the fact that current sex chromosomes derived from ancestral autosomes and have autosomal counterparts in species with nonhomologous sex chromosomes (Julien et al. 2012). Thus, we calculated ancestral expression levels based on the median value across the expression levels of one-to-one orthologs of XY gametologs in the platypus, chicken, and *A. carolinensis* (supplementary table 4*a*–*d*, Supplementary Material online).

We found that Y gametologs, by and large, showed an acute 3-fold decrease in expression compared with the ancestral expression levels, whereas X gametologs in males showed minor expression loss and, lastly, X gametologs in females were the only ones that maintained the ancestral expression levels (fig. 2*a*; Mann–Whitney *U* test against a distribution with fixed medians). This general pattern shows slight variations within the mammalian groups: Y gametologs show greater expression loss in rodents and afrotheria/xenarthra. Moreover, expression levels of X gametologs in both males and females are also depressed in primates and afrotheria/xenarthra (fig. 2*b*; Mann–Whitney *U* test against a distribution with fixed medians). As gene expression levels across vertebrate species are more strongly correlated with tissue specificity rather than with the species’ phylogeny (Brawand et al. 2011; Merkin et al. 2012; Cardoso-Moreira et al. 2019; Naqvi et al. 2019), we examined the evolution of XY gametologs in each tissue separately. We found that somatic tissues (brain, heart, kidney, and liver) follow the general pattern; that is, a 3-fold decrease in expression levels of Y gametologs, a minor expression loss of X gametologs in males and a stable, similar to the ancestral expression, for X gametologs in females (fig. 2*c*). In gonads, conversely, X gametologs also showed large expression reduction in males (fig. 2*c*), which is due to the transcriptional silencing of genes by meiotic sex chromosome inactivation mechanism (Turner 2007). We found that the general pattern of XY expression change is maintained, though we used maximum expression levels as metric instead of median values (supplementary fig. 7, Supplementary Material online).

Next, we analyzed that expression levels of Y gametologs across individual species and observed an important variation, though in several instances the values fluctuate around a 3-fold decrease in expression levels (median values across tissues) compared with the ancestral expression levels (fig. 2*d*; Mann–Whitney *U* test between groups). Interestingly, species showing the greatest Y expression loss are not found scattered across the species’ tree but they cluster in two groups (fig. 2*d*); three rodent species from the same superfamily (mouse, rat, and hamster; Muroidea) and the two afrotheria/xenarthra species (armadillo and tenrec). Moreover, the amount of expression loss of Y gametologs in placental species does not correlate with the species’ phylogeny (phylogenetic generalized least squares, PGLS, *P *>* *0.05; fig. 2*d*), species’ life-history traits (PGLS *P *>* *0.05; supplementary table 6, Supplementary Material online), or the number of conserved ancestral Y gametologs (PGLS *P *>* *0.05; figs. 1 and 2*c*).

Every species of placental mammals has been subjected to specific selection pressures during evolution, which, in turn, could have differentially affected the rate of expression decline of the Y chromosome. We reconstructed the hypothetical expression levels of Y gametologs in the ancestors of primates, rodents, laurasiatherios, and afrotherios/xenarthra. We found that the ancestor of primates and laurasiatherios showed ∼3-fold expression loss, the ancestors of rodents presented ∼4-fold expression loss, and the ancestor of afrotherios/xenarthra may have exhibited an acute ∼8-fold expression loss. These results suggest that, 90–100 Ma, the ancestor of placental mammals already featured an Y chromosome with less than half of its expression levels.

### Evolution of Tissue Specificity

**Fig. 3.**
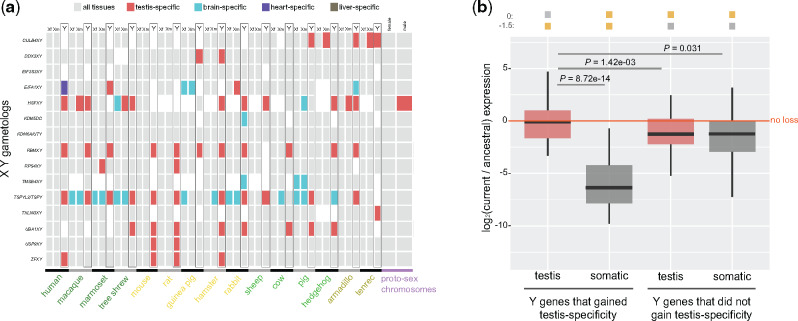
Patterns of tissue specificity of ancestral XY gametologs in 15 placental mammals. (*a*) TSI for X and Y gametologs in males and females. Estimates of TSI in the proto-sex chromosomes are also shown. Genes with a TSI <0.6 are colored in gray, whereas genes with a TSI > 0.6 are colored based on the tissue showing the highest expression level. Data from panda and mouse lemur were not included. (*b*) Boxplots representing the current/ancestral expression ratios of Y gametologs in testis and somatic tissues. Y gametologs were divided into two groups according to their testis specificity (genes with a testis-specificity index above 0.6 and genes with a testis-specificity index below 0.6). *HSFY* was excluded from the analyses. Ancestral expression levels were calculated for testis and somatic tissues separately. Significant differences (Mann–Whitney *U* test): Benjamin–Hochberg-corrected *P *<* *0.05 between groups and of ratios against a distribution with a fixed median of 0 (i.e., similar expression levels of current and ancestral states) or −1 (i.e., 2-fold loss in expression levels between current and ancestral states). Gray filled squares denote nonsignificant differences, whereas yellow filled squares denote significant differences. Error bars, maximum and minimum values, excluding outliers.

Next, we examined the expression evolution of Y gametologs across placental species. Theory predicts that Y genes could likely evolve male-beneficial functions (Burgoyne 1987; Chandley and Cooke 1994; Skaletsky et al. 2003) because they are, ultimately, male-specific genes. So, it was not surprising that Y gametologs in many species showed biased expression toward testis compared with both X gametologs and protosex chromosomes, which, generally, maintained ubiquitous expression across tissues (fig. 3*a*). We found that *HSFY* is the only Y gene with testis specificity that retained the expression pattern of the protosex chromosomes. In all other instances, remarkably, Y gametologs gained testis-biased expression due to an accelerated expression loss in somatic tissues compared with the testis (fig. 3*b*). That is, Y gametologs that evolved testis-specific expression did not lose expression in this tissue but lost significant expression levels in somatic tissues (fig. 3*b*; Mann–Whitney *U* test against a distribution with a fixed median and between groups). Alternatively, Y gametologs that did not evolve testis-biased expression showed similar expression loss in both somatic tissues and testis (fig. 3*b*; Mann–Whitney *U* test against a distribution with a fixed median and between groups). Although some Y genes acquired testis-biased expression in specific species (i.e., *CULB4Y* in pig, armadillo, and tenrec, *EIFA1Y* in marmoset, *ZFY* in human, mouse, rat, and hamster or *USP9Y* in mouse and rat; fig. 3*a*), our data suggest that at least *RBMY*, *TSPY*, and *UBA1Y* were already testis specific in the ancestor of placental mammals.

**Fig. 4.**
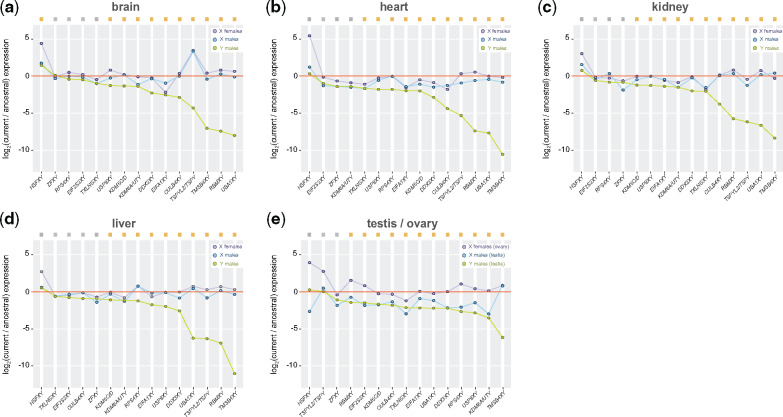
Expression levels of XY gametologs in different tissues. Dot plots representing median current/ancestral expression ratios of X and Y gametologs in males and females (*n* = 15–7 genes per gametolog; see fig. 1) in (*a*) brain, (*b*) heart, (*c*) kidney, (*d*) liver, and (*e*) gonads (testis/ovary). (*a*–*e*) Gametologs are ordered on the *X*-axis based on the Y gametologs values (decreasing order). Significant differences (Mann–Whitney *U* test): Benjamin–Hochberg-corrected *P *<* *0.05 of Y gametologs ratios against a distribution with a fixed median of 0 (i.e., no difference in current/ancestral expression levels). Gray filled squares denote nonsignificant differences, whereas yellow filled squares denote significant differences. Given that different tissues were available, data from panda and mouse lemur were not included.

At the gene level, we found that most Y gametologs have lost expression levels across tissues (fig. 4*a*; Mann–Whitney *U* test against a distribution with a fixed median), with *TMSB4Y* showing the greatest expression decline (fig. 4*a*–*e*). Remarkably, however, three Y gametologs (*HSFY*, *EIF2S3Y*, and *ZFY*) have consistently maintained the ancestral expression levels across tissues, suggesting that selection has been particularly keen on maintaining the expression level of these three genes (fig. 4*a*–*e*, genes in blue; Mann–Whitney *U* test against a distribution with a fixed median). In agreement with the results shown in figure 3, Y gametologs that have gained testis specificity in placental species (i.e., *TSPY*, *RBMY*, *UBA1Y*, and *CULB4Y*) exhibited considerable expression decline in somatic tissues (fig. 4*a*–*e*, genes in pink) but only minor expression loss in testis. In general, X gametologs appear to have more conserved expression levels in males and females (fig. 4*a*), with relatively higher expression in females compared with males, independently on the tissue (fig. 4*a*–*e*).

### Dosage Compensation Mechanisms

**Fig. 5.**
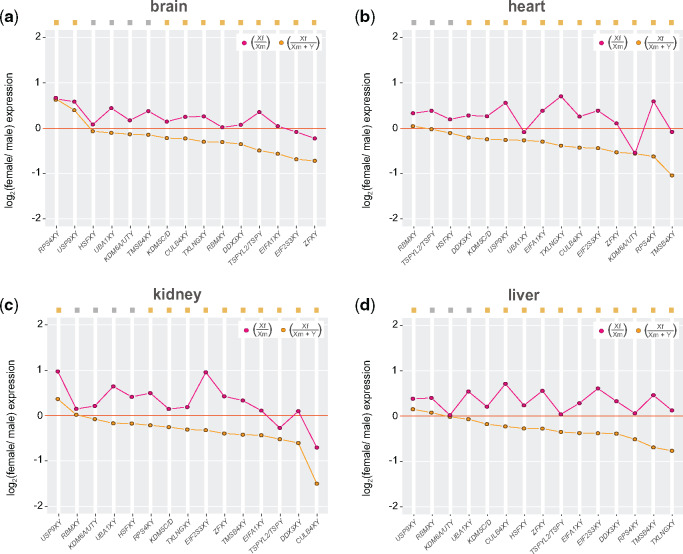
Female/male expression ratios of XY gametologs in different tissues. Dot plots representing median female/male expression ratios of X gametologs (pink dots), and X and Y gametologs (orange dots) in (*a*) brain, (*b*) heart, (*c*) kidney, and (*d*) liver; gonads were not analyzed because ovaries and testis represent different tissues. (*a*–*d*) Gametologs are ordered on the *X*-axis based on the Xf ÷ (Xm + Y) values (decreasing order). Formulas indicate the operations performed with X and Y gametologs to calculate the ratios. Significant differences (Mann–Whitney *U* test): Benjamin–Hochberg-corrected *P *<* *0.05 between groups (pink vs. orange dots; excluding male-biased *HSFXY*) and of Xf ÷ (Xm + Y) ratios against a distribution with a fixed median of 0 (i.e., similar expression levels of current and ancestral states). Gray filled squares denote nonsignificant differences, whereas yellow filled squares denote significant differences. Given that different tissues were available, data from panda and mouse lemur were not included.

We recurrently observed significantly higher expression of X gametologs in females compared with the X gametologs in males across our analyses (figs. 2, 4, and 5*a*–*d*). This result expands on previous notions that in human and mouse a majority of X gametologs escape X chromosome inactivation in females (Bellott et al. 2014; Tukiainen et al. 2017). Furthermore, we estimated that overall in placental mammals the additional expression of X gametologs in females corresponded to a +20%/+30% increase, which is remarkably similar to the measured extra expression (+30%) of X-linked genes escaping X chromosome inactivation in humans (Tukiainen et al. 2017). Interestingly, the combined expression of XY gametologs in males compared with the expression of X gametologs in females did not result in a balanced scenario where both sexes have similar expression levels (fig. 5*a*–*d*). Instead, we consistently observed greater expression levels of XY gametologs in males compared with females, regardless of the tissue that is analyzed (fig. 4*b*; Mann–Whitney *U* test against a distribution with a fixed median). In other words, despite the expression loss of Y genes, the sum of XY expression values is larger than the expression levels of the active X gametologs and the expression of the X gametologs escaping X chromosome inactivation. This observation strongly suggests that expression levels of X gametologs have also decline in both sexes. The pattern of male-biased expression levels for XY gametologs was also noted in humans across a variety of tissues (Tukiainen et al. 2017).

**Fig. 6.**
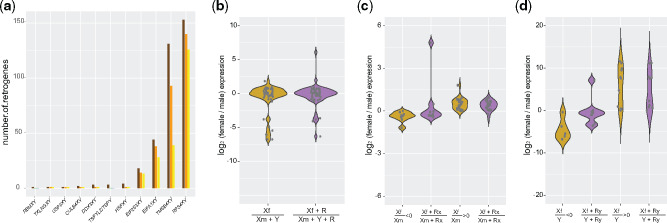
Expression levels of XY-derived retrogenes. (*a*) The number of XY-derived retrogenes. Brown bars show the total number of XY-derived retrogenes, orange bars show retrogenes with *d*_S_ < 1, and yellow bars show retrogenes with *d*_S_ < 1 and TPM > 1. (*b*) Violin plots representing the expression ratios of X gametologs in females compared with the combined expression of X and Y gametologs in males. Purple violin plot incorporates the expression of XY-derived retrogenes (denoted by the letter *R* in the formula). (*c*) Same as in (*b*) but for X gametologs and X-derived retrogenes. Gametologs were divided into two groups depending on whether Xf/Xm ratios were above or below zero. Purple violin plot incorporates the expression of X-derived retrogenes (denoted by the letters *Rx* in the formula). (*d*) Same as in (*b*) but for X gametologs in females, Y gametologs in males and Y-derived retrogenes. Gametologs were divided into two groups depending on whether Xf/Y ratios were above or below zero. Purple violin plot incorporates the expression of Y-derived retrogenes (denoted by the letters *Ry* in the formula). (*b*–*d*) Significant differences (Mann–Whitney *U* test) between groups. Median expression levels across tissues (TPM) were used for XY gametologs; cumulative expression was considered for retrogenes. Data from panda, mouse lemur, and hedgehog were not included.

Retrogenes allow genes to acquire new genomic locations, change tissue specificity (Carelli et al. 2016), or escape specific molecular processes such as X chromosome inactivation (Hurst et al. 2015). In particular, it has been proposed that gene duplications, such as retrogenes, could compensate for the loss of Y genes (Hughes et al. 2015) or compensate gene dosage between sexes (Andres et al. 2008) by balancing the expression decline of Y gametologs. Thus, we screened the genomes of placental mammals for retrogenes that derived from XY gametologs. We considered for further analyses only those retrogenes under purifying selection (*d*_S_ < 1) and showing active transcription (TPM > 1) (Carelli et al. 2016). Four XY gametologs (*RPS4XY*, *TMSB4XY*, *EIFA1XY*, and *EIF2S3XY*) comprised ∼95% of the total number of retrogenes (fig. 6*a*). We found that XY-derived retrogenes are lowly expressed (supplementary table 4*a*–*d*, Supplementary Material online) and expression levels of parental XY gametologs remained similar between males and females regardless of whether the expression of retrogenes was taken into account or not considered at all (fig. 6*b*), suggesting that retrogenes are expressed at similar levels in both sexes and do not overcompensate Y expression specifically in males. We observed similar results when we limited the analyses to the expression levels of parental X gametologs and X-derived retrogenes (fig. 6*c*) or parental Y gametologs and Y-derived retrogenes (fig. 6*d*). These results indicate a minor role of retrogenes in dosage compensation.

## Discussion

In this study, we explored and expanded the knowledge of the evolution of XY gametologs, particularly gene expression evolution, by analyzing species from the four major groups of placental mammals. We focused on ancestral XY gametologs that stopped recombination before the radiation of placental mammals; other recent studies investigated the variability of ampliconic families (Brashear et al. 2018; Vegesna et al. 2019). Our results suggested that the ancestor of placental mammals already presented a limited set of Y-linked genes. Some Y gametologs show remarkable conservation across placental species. These genes code for proteins with functions in transcription regulation or during spermatogenesis: *SRY* is the master-sex regulator of testis development (Li et al. 2014); *ZFY* is necessary for spermatogenesis (Nakasuji et al. 2017) and could be a key regulator of genome-wide dosage effects together with *ZFX* (Raznahan et al. 2018); *RBMY*, *TSPY*, *DDX3Y, USP9Y*, and *EIF2S3Y* are essential for spermatogenesis (Colaco and Modi 2018); finally, *UTY* is important in tumor suppression (Gozdecka et al. 2018). Interestingly, in species such as the spiny rat *Tokudaia muenninki* (Murata et al. 2016) and the mole vole *Ellobius* (Mulugeta et al. 2016) that lost the placental Y chromosome, some of the eight conserved Y gametologs moved from the placental Y chromosome onto the new sex chromosomes before the placental Y chromosome was lost (*ZFY*, *EIF2S3Y*, *TSPY*, *UTY*, *DDX3Y*, *USP9Y* in *T. muenninki*, and *ZFY*, *EIF2S3Y*, and *USP9Y* in *Ellobius* species). These results indicate that retention of these remarkably conserved Y-linked genes is more important than preserving entire sex chromosomes.

Previous work examined the expression patterns of X- and Y-linked genes in primates and rodents (Lingenfelter et al. 2001; Xu and Disteche 2006; Isensee et al. 2008; Xu et al. 2008; Wilson Sayres and Makova 2013; Cortez et al. 2014; Slavney et al. 2016; Vegesna et al. 2019; Godfrey et al. 2020). Some of these studies already reported that Y chromosomes in placental mammals had lost expression output. Nevertheless, our data allowed to examine the evolution of Y expression across placental groups and indicated that Y gametologs already showed significant expression decline in the ancestor of placental mammals and testis-specific genes. The reduction in expression levels could have originated through a gradual decaying process or by a swift expression decline followed by long-lasting expression stasis. We found that the expression of Y chromosomes is similar in most placental species, which would favor an evolutionary model where Y chromosomes followed swift expression decline and are currently under expression stasis. Rodents from the superfamily Muroidea and the two species from afrotheria/xenarthra presented greater Y expression loss, which could result from accelerated group-specific Y decline. Moreover, we found that three Y gametologs have minor expression loss in placental species. It would appear that these Y genes have successfully purged deleterious mutations from their promoter regions and their functions and haploinsufficiency are probably crucial. One of these genes is *ZFY*, a potential genome-wide dosage effects regulator along with *ZFX* (Raznahan et al. 2018). A second gene is *EIF2S3Y*, which is essential for spermatogenesis and embryogenesis (Mazeyrat et al. 2001; Yamauchi et al. 2014). The third gene is *HSFY*, which is also critical for spermatogenesis (Colaco and Modi 2018). Furthermore, we also uncovered that accelerated expression loss in somatic tissues compared with testis expression levels has been the main mechanism that allowed Y-linked genes to gain testis specificity, as also reported in Cortez et al. (2014), and, likely, specialized in relevant functions during spermatogenesis (Colaco and Modi 2018; Liu 2019). Lastly, we found little evidence relating Y expression loss enabling Y gene loss and that retrogenes, in general, compensate for either expression or gene losses.

We considered that the haploinsufficient nature of XY gametologs (Bellott et al. 2014; Cortez et al. 2014) would be incompatible with the expression decline of Y gametologs because males, eventually, would lack sufficient proteins to maintain cellular homeostasis. One potential solution to this problem could have been to increase the expression levels of the X chromosome in males. However, X genes in placental mammals have reached maximum expression levels (Hurst et al. 2015). The alternative was to coordinate the expression of Y-linked genes with that of X-linked genes. Recent work supports this idea of active cross-regulation between X and Y chromosomes regarding gene expression levels (Raznahan et al. 2018). This cross-regulation between sex chromosomes has the potential to explain that X gametologs lost expression output as a consequence of Y expression decay. Data retrieved from humans (Tukiainen et al. 2017), and the results from this study, indicated that expression levels of XY gametologs in males are slightly higher than the combined expression levels of X gametologs in females. So, it would appear that XY gametologs are not perfectly balanced in placental mammals. This premise, however, assumes that XY gametologs show complete functional redundancy, which is likely not the case. Although X and Y gametologs may act on similar cellular processes or interact with similar protein partners, Y gametologs have likely evolved specialized functions and affinities, for example, in testis-related functions. The available experimental data support this hypothesis given that in transgenic mice, only the overexpression of *SOX3* and *EIF2S3X* could functionally replace *SRY* and *EIF2S3Y*, respectively (Sutton et al. 2011; Yamauchi et al. 2016). Moreover, it has been shown that *UTY* has lower demethylase activity compared with *UTX*, owing to point substitutions affecting substrate binding (Walport et al. 2014; Gozdecka et al. 2018; Gazova et al. 2019). It is likely, therefore, that other Y genes also exhibit diminished activities caused by the accumulation of point substitutions. So, an interesting hypothesis worth studying in future work is that the extra expression of Y gametologs compensates for the reduced Y activity/affinity. Further experiments will be required to test this hypothesis. Interestingly, in a recent study, it was found that Y gametologs can show increased expression levels in specific tissues in humans (Godfrey et al. 2020). So, the mechanisms that maintain XY dosage between sexes and the general pattern of Y expression loss we observed across mammals can be reversed to meet yet unknown tissue-specific protein requirements.

## Ethics Approval and Consent to Participate

ERC Ethics Screening Panel (associated with ERC Consolidator Grant 615253) and ethics committee in Lausanne (authorization 504/12).

## Supplementary Material

Supplementary data are available at *Genome Biology and Evolution* online.

## Supplementary Material

evaa173_Supplementary_Data

## Notes

**Data deposition:** This project has been deposited at NCBI-SRA database (www.ncbi.nlm.nih.gov/sra; last accessed August 21, 2020) under the accession BioProject PRJNA580502. Y-linked sequences found in this study are available in the Supplementary Material online. Code and command line to apply the subtraction approach on male/female RNA-seq data are available in the Supplementary Material online.
