# Supplementary material for: Expression Evolution of Ancestral XY Gametologs across All Major Groups of Placental Mammals
Source: Genome Biol Evol. 2020 Aug 13;12(11):2015–28. doi: 10.1093/gbe/evaa173 (PMC7674692; doi:10.1093/gbe/evaa173)
Supplement: evaa173_Supplementary_Data [file evaa173_supplementary_data.zip › Supplementary Figures 1-7.pdf]

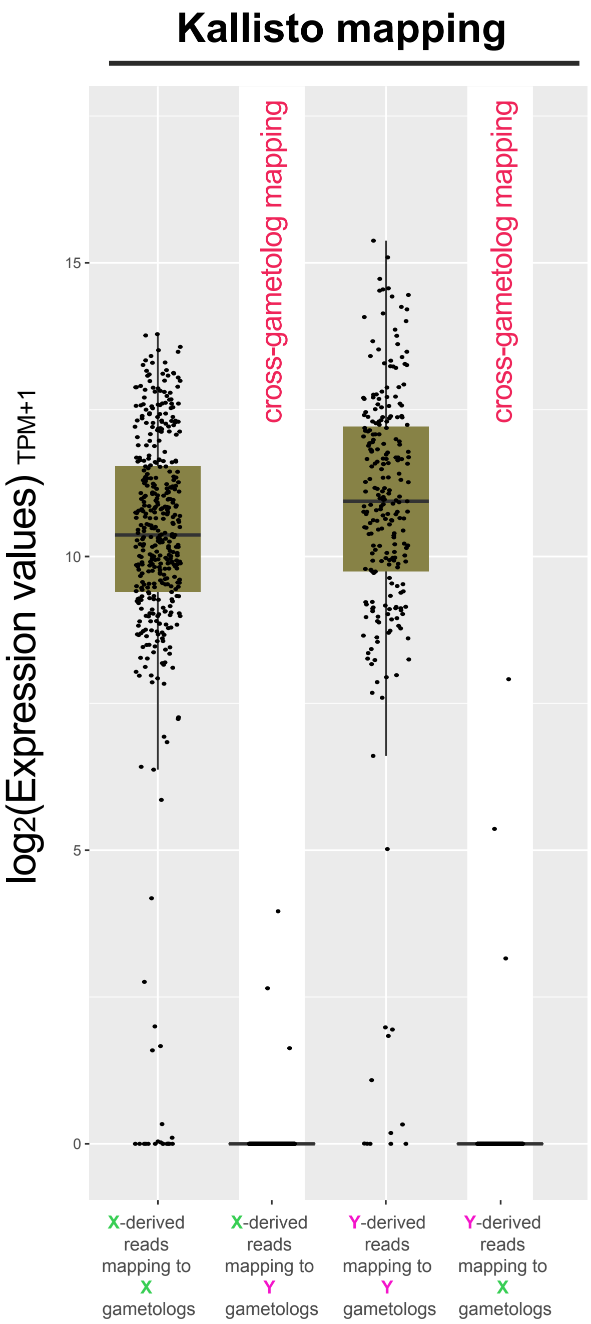

Supplementary Figure 1. Low incidence of cross-gametolog mapping observed after mapping X-derived or Y-derived *in sillico* generated reads to the species' transcriptomes using Kallisto.

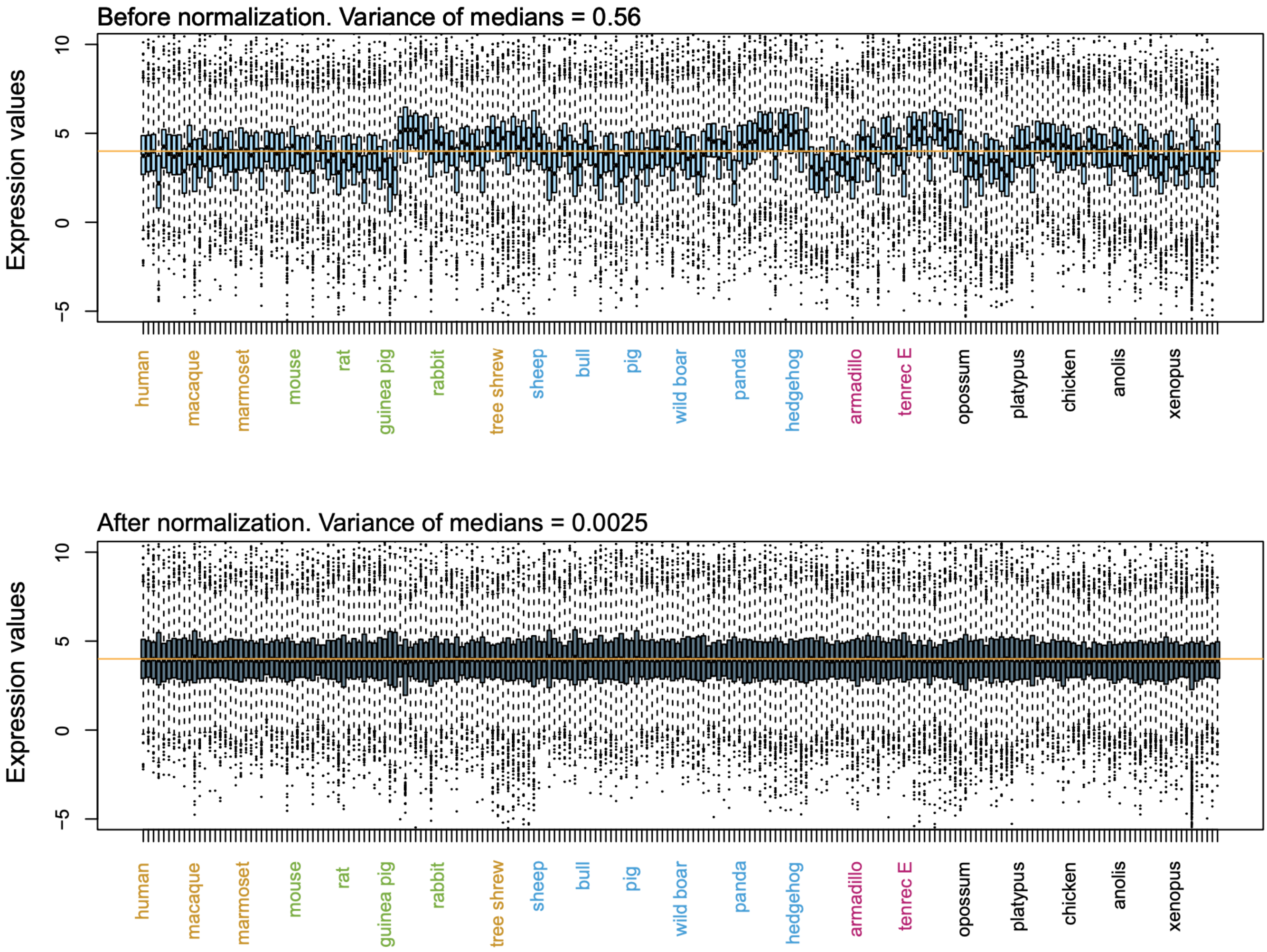

Supplementary Figure 2. Expression values of 1:1 orthologous genes expressed in all tissues before and after the re-scaling normalization procedure. The general variance across samples is largely reduced after the normalization.

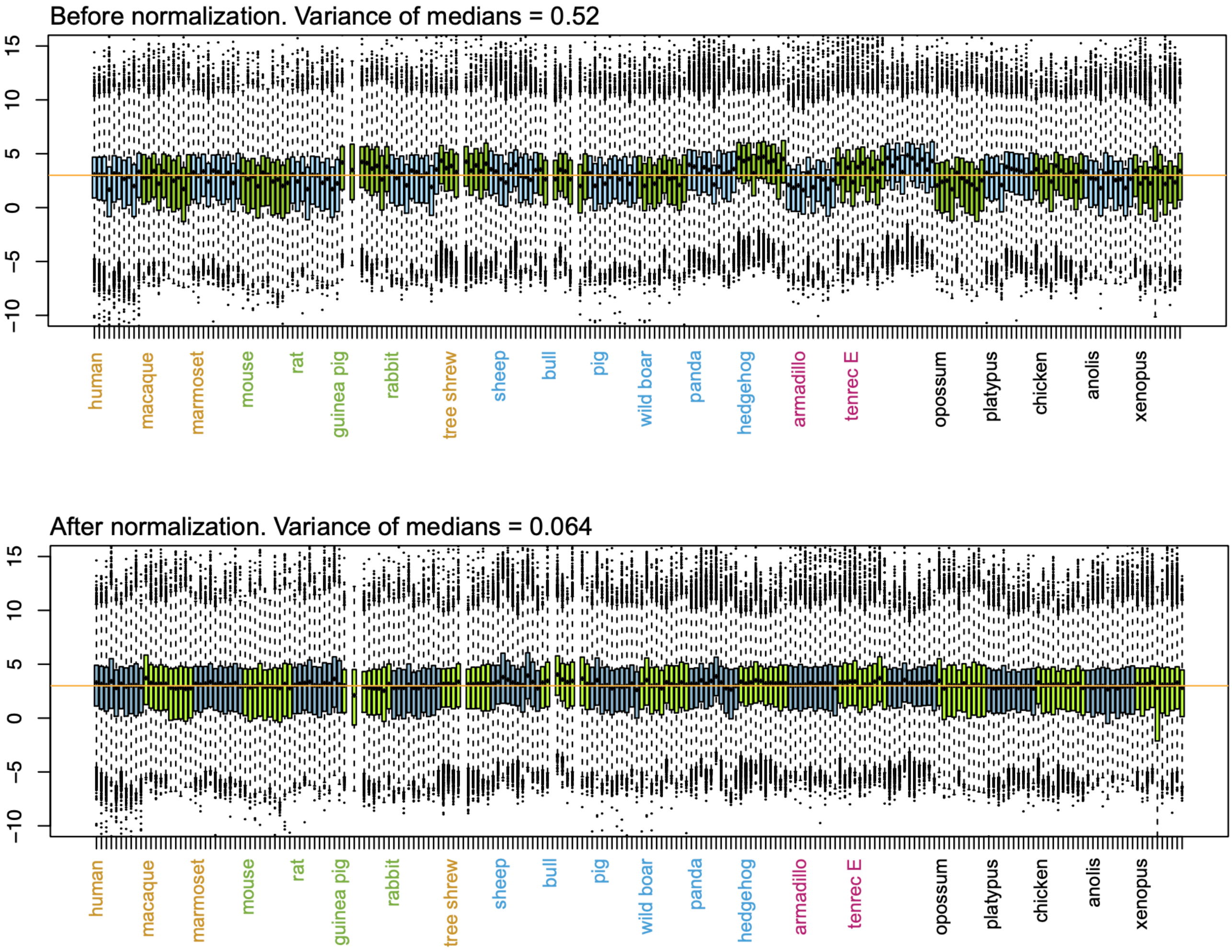

Supplementary Figure 3. Expression values of all genes across samples before and after the re-scaling normalization procedure. The general variance across samples is largely reduced after the normalization.

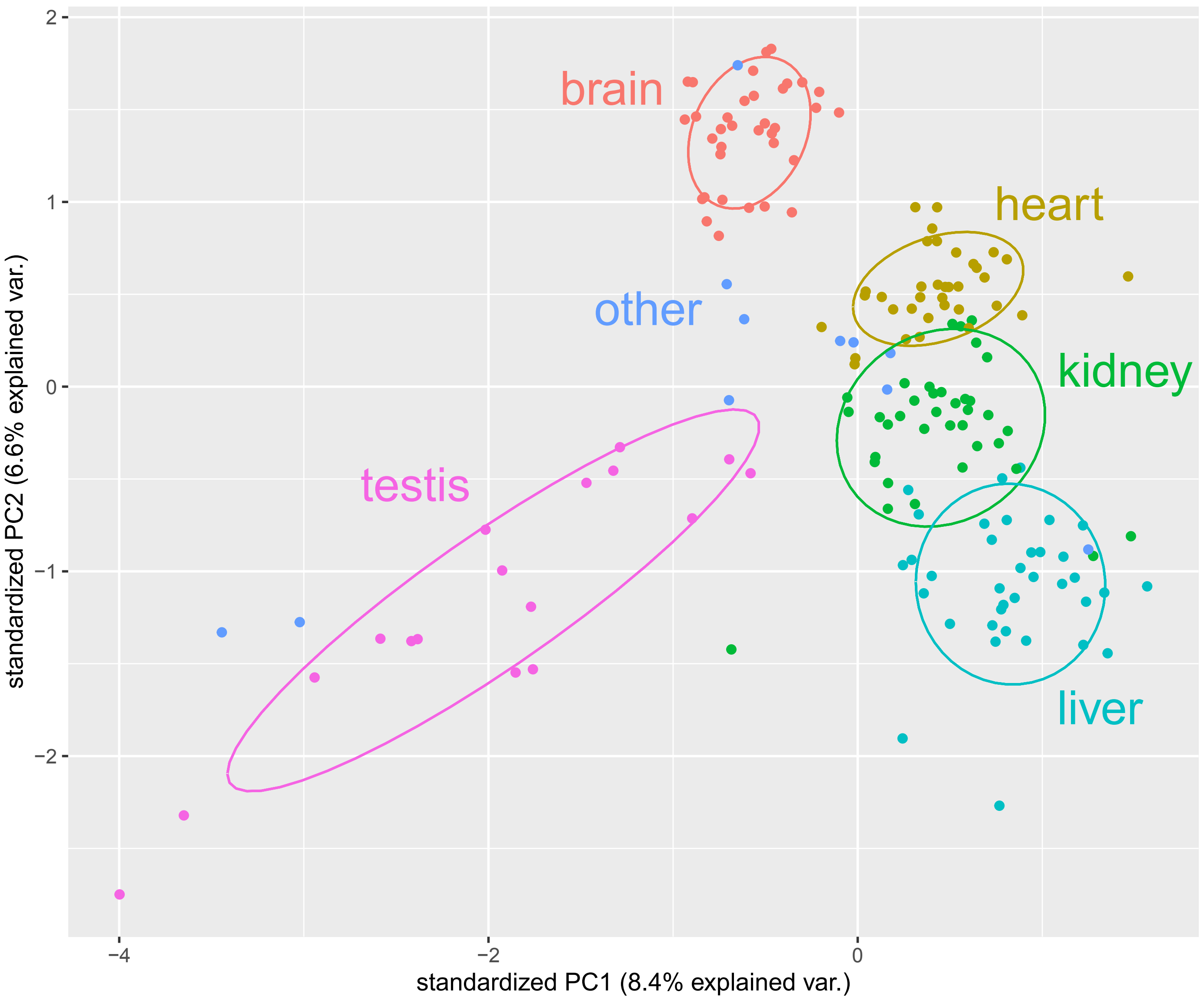

Supplementary Figure 4. PCA using expression levels of 1-to-1 ortholog genes in somatic and gonad tissues of placental species; samples cluster by tissue and not by experiment. The blue label *other* represents samples that were unique and came from somatic tissues that were not analyzed in other species, such as the stomach, intestine, tongue, etc. (see Supplementary Table 1).

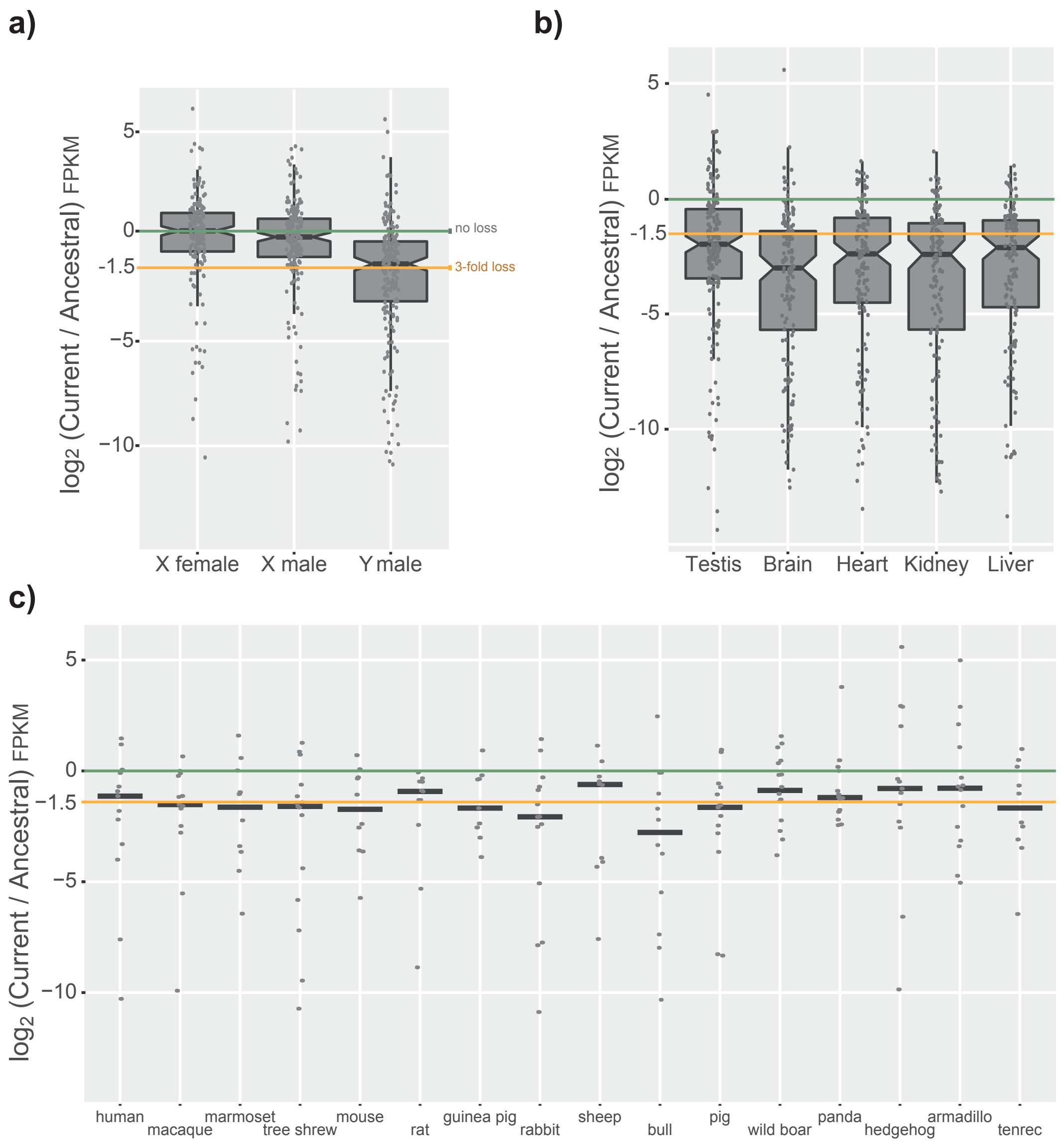

Supplementary Figure 5. Expression analyses of Y gametologs in placental species using FPKM values obtained from Hisat2 & Cufflinks.

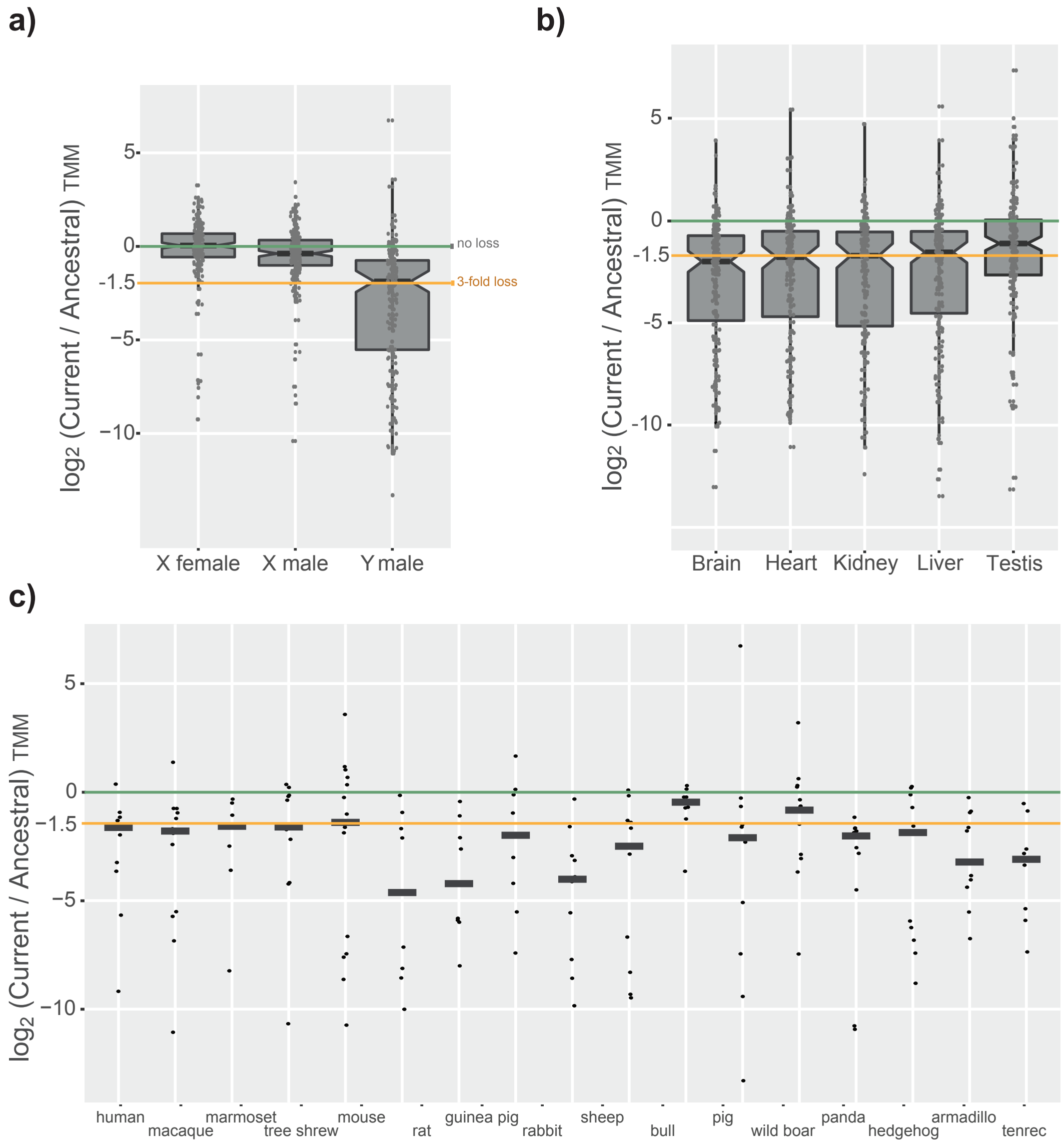

Supplementary Figure 6. Expression analyses of Y gametologs in placental species based on read counts from Kallisto; values were normalized using the TMM normalization in the EdgeR package.

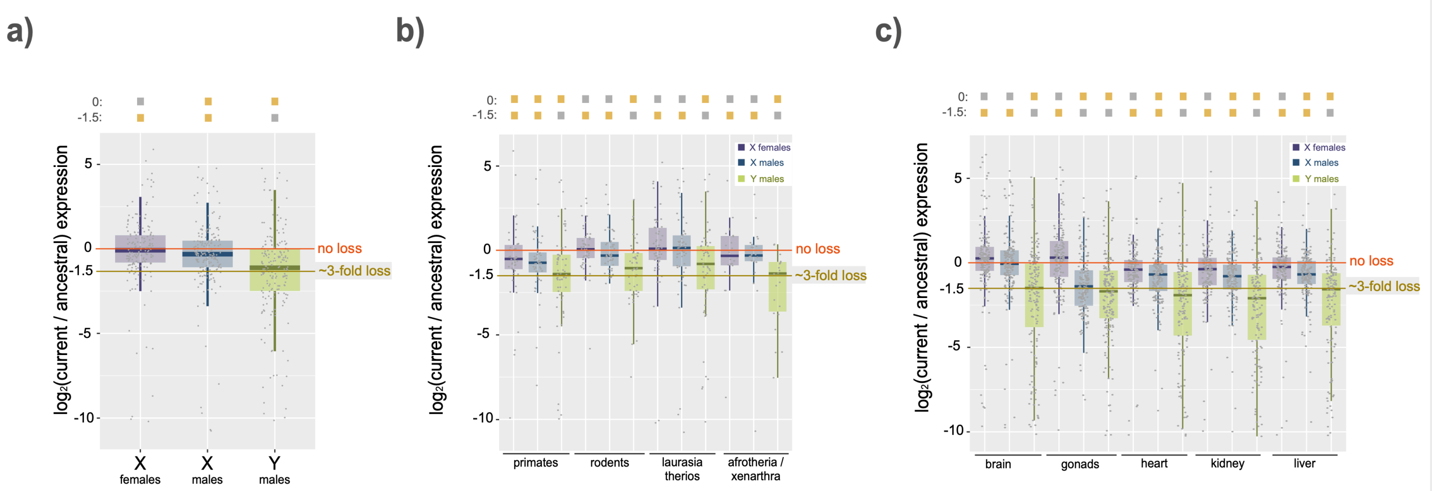

Supplementary figure 7. Expression levels of ancestral XY gametologs in 17 placental mammals (<u>Maximum expression across tissues</u>). a) Boxplots representing the current/ancestral expression ratio of X and Y gametologs in males and females (n = 200 genes). b) Boxplots representing the current/ancestral expression ratio of X and Y gametologs in males and females according to the phylogenetic group: Pr data from primates including tree shrew (n = 60 genes); Ro data from rodents including rabbit (n = 52 genes); La data from laurasiatheria (n = 65 genes); At data from armadillo and tenrec (n = 23 genes). c) Boxplots representing the current/ancestral expression ratio of X and Y gametologs in males and females according to the tissue: brain, gonads, heart, kidney, and liver. a-c) Significant differences (Mann–Whitney U test): Benjamin–Hochberg-corrected $P < 0.05$ of ratios against a distribution with a fixed median of 0 (i.e., similar expression levels of current and ancestral states) or -1 (i.e., 2-fold loss in expression levels between current and ancestral states). Gray filled squares denote nonsignificant differences, whereas yellow filled squares denote significant differences. Error bars, maximum and minimum values, excluding outliers. a-c) Maximum expression levels across tissues (TPM) were used.
