# Supplementary material for: Expression Evolution of Ancestral XY Gametologs across All Major Groups of Placental Mammals
Source: Genome Biol Evol. 2020 Aug 13;12(11):2015–28. doi: 10.1093/gbe/evaa173 (PMC7674692; doi:10.1093/gbe/evaa173)
Supplement: evaa173_Supplementary_Data [file evaa173_supplementary_data.zip › Supplementary.Material.Code.comand.line.obtainYsequences.pdf]

#####PERL SCRIPTS AND COMAND LINE USED TO PERFORM THE SUBTRACTION APPROACH INTENDED TO OBTAIN Y-LINKED CANDIDATES, ALIGN SEQUENCES, RUN CODEML AND GET THE SPECIE's ULTRAMETRIC TREE
#####2018-2019
#####SEE MORE DETAILS IN THE METHODS SECTION

###Transcriptome reconstruction of MALE or FEMALE tissues
#Done for male & female SpeciesA

Trinity-v2.8.5

```
Trinity --seqType fq  --min_contig_length 300 --max_memory 200G --output SpeciesA.Female_alltissues_trinity --trimmomatic --CPU 15 --full_cleanup --left SpeciesA.Female.Brain_R1.fastq.gz,SpeciesA.Female.Liver_R1.fastq.gz,SpeciesA.Female.Ovary_R1.fastq.gz --right SpeciesA.Female.Brain_R2.fastq.gz,SpeciesA.Female.Liver_R2.fastq.gz,SpeciesA.Female.Ovary_R2.fastq.gz

Trinity --seqType fq  --min_contig_length 300 --max_memory 200G --output SpeciesA.Male_alltissues_trinity --trimmomatic --CPU 15 --full_cleanup --left SpeciesA.Male.Brain_R1.fastq.gz,SpeciesA.Male.Liver_R1.fastq.gz,SpeciesA.Male.Testis_R1.fastq.gz --right SpeciesA.Male.Brain_R2.fastq.gz,SpeciesA.Male.Liver_R2.fastq.gz,SpeciesA.Male.Testis_R2.fastq.gz
```

###Hisat
#Done for male & female SpeciesA

hisat2-2.0.2

```
hisat2-build SpeciesA.Male_alltissues_trinity.Trinity.fasta SpeciesAMaleindex.hisat

hisat2-build SpeciesA.Female_alltissues_trinity.Trinity.fasta SpeciesAFemaleindex.hisat
```

```
#Perl script
@data= qw(
SpeciesA.Male.Testis_R1
SpeciesA.Male.Testis_R2
SpeciesA.Male.Brain_R1
SpeciesA.Male.Brain_R2
SpeciesA.Male.Liver_R1
SpeciesA.Male.Liver_R2
);
```

```
foreach $i (0..$#data) {
   print "$i of ",$#data," => Male: $data[$i]\n";
       system("hisat2 -q  -p 5 --rna-strandness R -x
SpeciesAFemaleindex.hisat -U $data[$i].fastq -S $data[$i].sam --un
$data[$i].unmapped");
}


###build 35kmer female library
#Done for female SpeciesA
#Perl script

@Female ="";
@Female = qw(
SpeciesA.Female.Ovary_R1.fastq
SpeciesA.Female.Ovary_R2.fastq
SpeciesA.Female.Brain_R1.fastq
SpeciesA.Female.Brain_R2.fastq
SpeciesA.Female.Liver_R1.fastq
SpeciesA.Female.Liver_R2.fastq
);

%fkmer=""; $ftramp=0; $mas=0;
foreach $i (0..$#Female) {
    print "$Female[$i]\n"; $tic=0; $mas=0;

  #system("gunzip $male[$i].gz");

    open (OUT,"$Female[$i]") || die;
        while(<OUT>) {
           chomp;
            $mas++;
            if ($mas==2) {
                $l= length($_);
            foreach $k (0..$l-35) {
                $as = substr($_,$k,35);
                $ax = substr($_,$k,2);
                  if ($as=~/N/) { }
                  else {
                    if ($ax eq "AG") {
                        $fkmer{$as}++;
                            if ($fkmer{$as} == 1) {
                            $ftramp++;
                        }
                    }
                }
                }
            }
           if ($mas==4) {
                $mas=0;
```

```
            }
        }
        close(OUT);
        print "$ftramp\n";
}

open (SAL,">SpeciesA.Female.35kmers.txt");
        print "output\n";
        foreach $h (keys %fkmer) {
        if ($fkmer{$h} > 49) {
            $numero++;
                    print SAL ">kmero$numero\n$h\n";
            }
        }
close(SAL);


###get fastq to fasta
#Done for male SpeciesA
#Perl script

@data =qw(
SpeciesA.Male.Testis_R1.unmapped
SpeciesA.Male.Testis_R2.unmapped
SpeciesA.Male.Brain_R1.unmapped
SpeciesA.Male.Brain_R2.unmapped
SpeciesA.Male.Liver_R1.unmapped
SpeciesA.Male.Liver_R2.unmapped
);

foreach $k (0..$#data) {
  print "$data[$k]\n";
    system ("fastq_to_fasta -v -i $data[$k] -o $data[$k].fa");
    system("bowtie-build $data[$k].fa $data[$k].fa");
}


###Bowtie unmapped vs 35kmer library
#Done for male SpeciesA
#Perl script

@data1 = qw(
SpeciesA.Male.Testis_R1.unmapped.fa
SpeciesA.Male.Testis_R2.unmapped.fa
SpeciesA.Male.Brain_R1.unmapped.fa
SpeciesA.Male.Brain_R2.unmapped.fa
SpeciesA.Male.Liver_R1.unmapped.fa
SpeciesA.Male.Liver_R2.unmapped.fa
);
```

```
@kmer = qw(
SpeciesA.Female.35kmers.txt
SpeciesA.Female.35kmers.txt
SpeciesA.Female.35kmers.txt
SpeciesA.Female.35kmers.txt
SpeciesA.Female.35kmers.txt
SpeciesA.Female.35kmers.txt
);



$read=0;
foreach $i (0..$#data1) {
        print "$data1[$i] ... $kmer[$i]\n";
        system ("bowtie -t -a -v 0 -p 5 -f --fullref --suppress 5,6
$data1[$i] $kmer[$i] >$data1[$i].35kmers.sam");
}




###Select male-specific reads
#Done for male SpeciesA
#Perl script

@data = qw(
SpeciesA.Male.Testis_R1.unmapped.fa.35kmers.sam
SpeciesA.Male.Testis_R2.unmapped.fa.35kmers.sam
SpeciesA.Male.Brain_R1.unmapped.fa.35kmers.sam
SpeciesA.Male.Brain_R2.unmapped.fa.35kmers.sam
SpeciesA.Male.Liver_R1.unmapped.fa.35kmers.sam
SpeciesA.Male.Liver_R2.unmapped.fa.35kmers.sam
);

@data2 = qw(
SpeciesA.Male.Testis_R1.unmapped.fa
SpeciesA.Male.Testis_R2.unmapped.fa
SpeciesA.Male.Brain_R1.unmapped.fa
SpeciesA.Male.Brain_R2.unmapped.fa
SpeciesA.Male.Liver_R1.unmapped.fa
SpeciesA.Male.Liver_R2.unmapped.fa
);

open(HUL,">log");

foreach $i (0..$#data) {
%mem="";
@w="";
        print "Analyzing: $data[$i]\n";
        open(OUT,"$data[$i]") || die;
```

```
                while(<OUT>) {
                        chomp;
                        split("\t");
                        @w=split("\t");
                        $mem{$w[2]} = 2; $tit++; if ($tit<11) {print
"$w[2]\n";}
                }
                close(OUT);

open(SAL,">$data[$i].femfiltered.fa");
$tot=0; $tut=0; $tem=1;
open (CAL, "$data2[$i]") || die;
        while(<CAL>) {
                chomp;
                if (/>/) {
                        $name=$_;
                        s/>/$rien/g;
                        $tot++;
                                if ($mem{$_} == 2) {
                                        $tem=2;
                                        $tut++;
                                }
                }
                else {
                        if($tem==1) {
                                print SAL "$name\n$_\n";
                        }
                        $tem=1;
                }
        }
        close(CAL);
        close(SAL);

        print "TOTAL_READS:$tot\tREMOVED_READS:$tut\n";
        print HUL "TOTAL_READS:$tot\tREMOVED_READS:$tut\n";

}
close(HUL);


###Match paired reads
#Done for male SpeciesA
#Perl script

@source = qw(
SpeciesA.Male.Testis_R1.unmapped.fa.35kmers.sam.femfiltered.fa
SpeciesA.Male.Brain_R1.unmapped.fa.35kmers.sam.femfiltered.fa
SpeciesA.Male.Liver_R1.unmapped.fa.35kmers.sam.femfiltered.fa
);
```

```
foreach $T (0..$#source) {
        $x = $source[$T];
        $x =~s/_R1/_R2/g;
        print "$T       $source[$T]     $x\n";
        %nn=""; %seq1=""; %seq2=""; $g=0; $g2=0;

        print "load 1 :: $source[$T]\n";
        open(OUT,"$source[$T]") || die;
                while(<OUT>) {
                        chomp;
                        if (/>/) {
                                @name=split(" ");
                                $nn{$name[0]}++; $g1++; if ($g1<6)
{print "$name[0]\n";}
                        }
                        else {
                                $seq1{$name[0]} = $_;
                        }

                }
                close(OUT);

        print "load 2 :: $x\n";
        open(OUT,"$x") || die;
                while(<OUT>) {
                        chomp;
                        if (/>/) {
                                @name=split(" ");
                                $nn{$name[0]}++; $g2++; if ($g2<6)
{print "$name[0]\n";}
                       }
                        else {
                                $seq2{$name[0]} = $_;
                        }
                }
                close(OUT);

        $remaing=0; $remaingT=0;
        print "output\n";
        open (SAL,">$source[$T].paired");
        open (SOL,">$x.paired");
        foreach $k (sort keys %nn) {
                if ($nn{$k} == 2) {
                         $tric=1;
                         $remaingT++;
                         if ($seq1{$k}=~/N/) {
                                $tric++;
                         }
                         if ($seq2{$k}=~/N/) {
                                $tric++;
```

```
                    }
                    if ($tric==1) {
                           print SAL "$k".".1\n$seq1{$k}\n";
                           print SOL "$k".".2\n$seq2{$k}\n";
                           $remaing++;
                   }
            }
            if ($nn{$k} > 2) {
                   print "WIERD::  $k $nn{$k}\n";
            }
     }
     close(SAL);
     close(SOL);
     print "done... reads:$remaing...with N's:$remaingT\n";
}


###TRINITY with MALE-specific reads
#Done for male SpeciesA

Trinity --seqType fa --min_contig_length 300 --left
SpeciesA.Male.All_Organs_R1.paired.fa --right
SpeciesA.Male.All_Organs_R1.paired.fa --max_memory 200G --output
SpeciesA.Male.All_Organs_R1-2.paired_trinity --CPU 15 --full_cleanup


##Select transcripts longer than 100-nt
#Done for male SpeciesA
#Perl script

open(SAL,">SpeciesA.Male.All_Organs_R1-2.paired_1000pb_trinity.Trinity
.fasta");
open
(OUT,"SpeciesA.Male.All_Organs_R1-2.paired_trinity.Trinity.fasta") ||
die;
    while(<OUT>) {
        chomp;

         if (/>/) {
                 @lista = split(" ",$_);
                 $r=0;
                 $lista[1]=~s/len=//g;
                         if ($lista[1] >= 1000) {
                                 $r=1;
                         }
                 if($r==1) {
                         print SAL "$_\n";
                 }
           }
         else {
```

```
                if($r==1) {
                        print SAL "$_\n";
                }
        }
}
close(OUT);
close(SAL);
```

###Blastn to locate X gametolgs
#Done for male SpeciesA
makeblastdb -in SpeciesA.Female_alltissues_trinity -dbtype nucl

blastn -task blastn -query SpeciesA.Male.All_Organs_R1-2.paired_1000pb_trinity.Trinity.fasta -db SpeciesA.Female_alltissues_trinity -out MalevsFemaletranscripts.out -dust no -evalue 0.00001 -outfmt 6 -num_threads 15

###Change format of Trinity output
#Done for male SpeciesA
#Perl script

```
open(IN,"SpeciesA.Male.All_Organs_R1-2.paired_trinity.Trinity.fasta")
|| die;
        while(<IN>) {
                chomp;
        #       $mas++;
                if (/>/) {
                $mas++; $r=0;
                        if ($mas==1) {
                                s/len=/$rien/g;
                                @w=split(" ");
                                if ($w[1] < 401) {
                                 if($mas > 6401) {
                                        print ">Trinity_".$mas."_".
$w[1]."\n";

                                        $r=1;
                                        }
                                }
                        }
                        if ($mas>1) {
                              s/len=/$rien/g;
                              @w=split(" ");
                              if ($w[1] < 401) {
                                 if($mas > 6401) {
                                        print "\n>Trinity_".
$mas."_".$w[1]."\n";
                                $r=1;
                                  }
```

```
                        }
                }
        }
        else {
                if ($r==1) {
                        print "$_";
                }
        }
}
print "\n";
close(IN);
```

###Blastn male transcripts vs. female genome
#Done for male SpeciesA

```
blastn -task blastn -query
SpeciesA.Male.All_Organs_R1-2.paired_trinity.Trinity.fasta.lign -db
EHF.R1.R2.fasta -out EH.maletrinity.femalegenome.txt -dust no -evalue
0.00001 -outfmt 6 -num_threads 15 -num_alignments 10000

blastn -task blastn -query
SpeciesA.Male.All_Organs_R1-2.paired_trinity.Trinity.fasta.lign -db
EHM.R1.R2.fasta -out EH.maletrinity.malegenome.txt -dust no -evalue
0.00001 -outfmt 6 -num_threads 15 -num_alignments 10000
```

###Get coverage Female genome
#Done for male SpeciesA
#Perl script

```
open(IN,"SpeciesA.Male.All_Organs_R1-2.paired_trinity.Trinity.fasta.li
gn");
        while(<IN>) {
                        chomp;
                                if (/>/) {
                                                s/>/$rien/g;
                                                $as=$_;
                                }
                                else {
                                                $mem{$as} = $_;
                                }
        }
        close(IN);

                open(IN,"EH.maletrinity.femalegenome.txt");
                        while(<IN>) {
                                        chomp;
                                        @w=split("\t");
                                                if ($w[2] > 98) {
                                                                if
($w[3] > 49) {
```

```
                $len2 = $w[7] - $w[6] + 1;

                $seq2=""; $seq2 = "X" x $len2;

                substr($mem{$w[0]},$w[6]-1,$len2) = $seq2;
                                                            }
                                                }
                        }
                        close(IN);

                        foreach $i (keys %mem) {
                                        @w=split("_",$i);
                                        $mem{$i}=~s/X/$rien/g;
                                        $len3 = length($mem{$i});
                                        $oper1 = ($len3*100)/$w[2];
                                                if ($oper1 > 50) {

print "$i\n$mem{$i}\n";
                                                }
                        }

###Get coverage Male genome
#Done for male SpeciesA
#Perl script

open(IN,"SpeciesA.Male.All_Organs_R1-2.paired_trinity.Trinity.fasta.li
gn");
        while(<IN>) {
                        chomp;
                                if (/>/) {
                                                s/>/$rien/g;
                                                $as=$_;
                                }
                                else {
                                                $mem{$as} = $_;
                                }
        }
        close(IN);

                open(IN,"EH.maletrinity.malegenome.txt");
                        while(<IN>) {
                                        chomp;
                                        @w=split("\t");
                                                if ($w[2] > 98) {
                                                            if
($w[3] > 49) {

                $len2 = $w[7] - $w[6] + 1;
```

```
                $seq2=""; $seq2 = "X" x $len2;

                substr($mem{$w[0]},$w[6]-1,$len2) = $seq2;
                                                                }
                                                }
                        }
                        close(IN);

                        foreach $i (keys %mem) {
                                        @w=split("_",$i);
                                        $mem{$i}=~s/X/$rien/g;
                                        $len3 = length($mem{$i});
                                        $oper1 = ($len3*100)/$w[2];
                                                if ($oper1 > 50) {

print "$i\n$mem{$i}\n";

                                                }
                        }
###Filter 1
#Done for male SpeciesA
#Perl script

$file = "EH.maletrinity.malegenome.txt";
open(OUT,">$file.filtered");
open(IN,"$file") || die;
        while(<IN>) {
                chomp;
                @w=split("\t");
                        if ($w[2] >= 98) {
                                if($w[3] >= 40) {
                                        print OUT "$_\n";
                                }
                        }
        }
        close(IN);
        close(OUT);



###Filter 2
#Done for male SpeciesA
#Perl script

open(IN,"SpeciesA.Male.All_Organs_R1-2.paired_trinity.Trinity.fasta.li
gn") || die;
        while(<IN>) {
                chomp;
                if(/>/) {
                        s/>/$rien/g;
                        $as=$_;
```

```
                        @w=split("_",$as);
                        $seq0 = "X" x $w[2];
                        $mem1{$as} = $seq0;
                        $mem2{$as} = $seq0;
                }
        }
        close(IN);
print "upload done\n";
open(IN,"EH.maletrinity.femalegenome.txt.filtered") || die;
        while(<IN>) {
                chomp;
                @w=split("\t");
                $len="";
                $len = $w[7] - $w[6] +1;
                $sec = "N" x $len;
                substr($mem1{$w[0]},$w[6],$len) = $sec;
        }
        close(IN);
print "substr done\n";

open(IN,"EH.maletrinity.malegenome.txt.filtered") || die;
        while(<IN>) {
                chomp;
                @w=split("\t");
                $len="";
                 $len = $w[7] - $w[6] +1;
                if ($mem2{$w[0]} ne $rien) {
                        $sec = "N" x $len;
                 substr($mem2{$w[0]},$w[6],$len) = $sec;
                 }
                 }
        close(IN);
print "substr done\n";

open(SAL, ">EH.maletrinity.cov");
foreach $i (sort keys %mem1) {
        $seq1 = $mem1{$i};
        $len1=""; $len11="";
        $len1 = length($seq1);
        $seq1 =~s/N/$rien/g;
        $len11 = length($seq1);
        $cov1 = ($len11*100)/$len1;

        $seq2 = $mem2{$i};
        $len2=""; $len22="";
        $len2 = length($seq2);
        $seq2 =~s/N/$rien/g;
        $len22 = length($seq2);
        $cov2 = ($len22*100)/$len2;
```

```
        print "$i\t$cov1\t$cov2\t$seq1\t$seq2\n";
        print SAL "$i\t$cov1\t$cov2\t$seq1\t$seq2\n";
}
close(SAL);


###Get specific transcript sequences
#Done for Y-linked SpeciesA
#Perl script

@data = qw(
TR108907--c2_g4_i
);

foreach $i (0..$#data) {
  open(IN,"SpeciesA.Male_trinity.Trinity.fasta") || die;
    while(<IN>) {
      chomp;
      if (/>/) {
        $r=1;
        if(/$data[$i]/) {
          print "$_\n";
          $r=2;
        }
      }
      else {
        if ($r==2) {
          print "$_\n";
        }
      }
    }
    close(IN);
}

###Alignment with PRANK
prank v.170427

prank -d=sequence_file -t=tree_file -o=output_file -f=output_format
-codon


###Concatenate individual alignments
#Done for Y-linked SpeciesA
#Perl script

@files = qw(
ATP2B1.anolis.out.best.fas
BTG1.anolis.out.best.fas
C12orf50.anolis.out.best.fas
CAND1.anolis.out.best.fas
```

```
FRS2.anolis.out.best.fas
MAN1.anolis.out.best.fas
PPP1R12A.anolis.out.best.fas
TNPO3.anolis.out.best.fas
UBE2H.anolis.out.best.fas
ZDHHC17.anolis.out.best.fas
);

foreach $i (0..$#files) {
  open(IN,"$files[$i]") || die;
    while(<IN>) {
      chomp;
      if (/>/) {
        $r=0;
          if (/anolis/) { $a = "anolis";}
          if (/Y/) { $a = "EH_Y";}
          if (/X/) { $a = "EH_X";}
          if (/pogona/) {$rx++; $a = "pogona";}
          if (/chicken/) { $a = "chicken";}
          if (/turtle/) { $a = "turtle";}
          if (/snake/) {$rr++; $a = "tiger_snake";}
          if (/tuatara/) { $a = "tuatara";}
          if (/human/) { $a = "human";}
          if (/mouse/) { $a = "mouse";}
          if (/opossum/) { $a = "opossum";}
          if (/xenopus/) { $a = "xenopus";}
      }
      else {
          $seq{$a}=$seq{$a}.$_;
          }
    }
    close(IN);
}

open (OUT,">concatenated");
foreach $j (sort keys %seq) {
  print "$j\n".length($seq{$j})."\n";
  print OUT ">$j\n$seq{$j}\n";
}
close(OUT);
print "$rx\n$rr\n";


###Control file to run codeml with concatenated alignment
      seqfile = concatenated
      outfile = concatenated.zero
      treefile = tree

        noisy = 0  * 0,1,2,3,9: how much rubbish on the screen
      verbose = 1  * 0: concise; 1: detailed, 2: too much
```

```
      runmode = 0  * 0: user tree;  1: semi-automatic;  2: automatic
                   * 3: StepwiseAddition; (4,5):PerturbationNNI; -2:
pairwise

      seqtype = 1  * 1:codons; 2:AAs; 3:codons-->AAs
    CodonFreq = 2  * 0:1/61 each, 1:F1X4, 2:F3X4, 3:codon table
*        ndata = 1
        clock = 0  * 0:no clock, 1:clock; 2:local clock;
3:CombinedAnalysis
       aaDist = 0  * 0:equal, +:geometric; -:linear,
1-6:G1974,Miyata,c,p,v,a
   aaRatefile = dat/jones.dat  * only used for aa seqs with
model=empirical(_F)
                   * dayhoff.dat, jones.dat, wag.dat, mtmam.dat, or
your own

        model = 1
                   * models for codons:
                       * 0:one, 1:b, 2:2 or more dN/dS ratios for
branches
                   * models for AAs or codon-translated AAs:
                       * 0:poisson, 1:proportional, 2:Empirical,
3:Empirical+F
                       * 6:FromCodon, 7:AAClasses, 8:REVaa_0,
9:REVaa(nr=189)

      NSsites = 0  * 0:one w;1:neutral;2:selection;
3:discrete;4:freqs;
                   * 5:gamma;6:2gamma;7:beta;8:beta&w;9:beta&gamma;
                   * 10:beta&gamma+1; 11:beta&normal>1;
12:0&2normal>1;
                   * 13:3normal>0

        icode = 0  * 0:universal code; 1:mammalian mt; 2-10:see below
        Mgene = 0
                   * codon: 0:rates, 1:separate; 2:diff pi, 3:diff
kapa, 4:all diff
                   * AA: 0:rates, 1:separate

    fix_kappa = 0  * 1: kappa fixed, 0: kappa to be estimated
        kappa = 2  * initial or fixed kappa
    fix_omega = 0  * 1: omega or omega_1 fixed, 0: estimate
        omega = .4 * initial or fixed omega, for codons or codon-based
AAs

    fix_alpha = 1  * 0: estimate gamma shape parameter; 1: fix it at
alpha
        alpha = 0. * initial or fixed alpha, 0:infinity (constant
rate)
       Malpha = 0  * different alphas for genes
```

```
        ncatG = 3  * # of categories in dG of NSsites models

        getSE = 0  * 0: don't want them, 1: want S.E.s of estimates
 RateAncestor = 1  * (0,1,2): rates (alpha>0) or ancestral states (1
or 2)

   Small_Diff = .5e-6
    cleandata = 1  * remove sites with ambiguity data (1:yes, 0:no)?
*  fix_blength = -1  * 0: ignore, -1: random, 1: initial, 2: fixed
        method = 0   * 0: simultaneous; 1: one branch at a time


* Genetic codes: 0:universal, 1:mammalian mt., 2:yeast mt., 3:mold
mt.,
* 4: invertebrate mt., 5: ciliate nuclear, 6: echinoderm mt.,
* 7: euplotid mt., 8: alternative yeast nu. 9: ascidian mt.,
* 10: blepharisma nu.
* These codes correspond to transl_table 1 to 11 of GENEBANK.

### Get ultrametric tree in R
library(maps)
library(phytools)
library(phylolm)
library(ape)

all.species.tree <- read.tree("SpeciesA/ds.tree.txt")

ctree <- chronos(all.species.tree)

plot(ctree)
edgelabels(ctree$edge.length, bg="black", col="white", font=2)
```
